# Systemic Administration of Tempol, a Superoxide Dismutase Mimetic, Augments Upper Airway Muscle Activity in Obese Zucker Rats

**DOI:** 10.3389/fphar.2022.814032

**Published:** 2022-02-09

**Authors:** Santhosh M. Baby, Lisa H. Tanner, Joseph F. Discala, Ryan B. Gruber, Yee-Hsee Hsieh, Stephen J. Lewis

**Affiliations:** ^1^ Department of Drug Discovery, Galleon Pharmaceuticals, Inc., Horsham, PA, United States; ^2^ Division of Pulmonary, Critical Care and Sleep Medicine, University Hospitals Case Medical Center, Case Western Reserve University, Cleveland, OH, United States; ^3^ Department of Pediatrics, Division of Pulmonology, Allergy and Immunology, School of Medicine, Case Western Reserve University, Cleveland, OH, United States; ^4^ Department of Pharmacology, School of Medicine, Case Western Reserve University, Cleveland, OH, United States

**Keywords:** Tempol, oxidative stress, diaphragm, genioglossus muscles, pharyngeal dilator muscle, upper airway, Zucker fat rats, obstructive sleep apnea

## Abstract

Obstructive sleep apnea (OSA) is characterized by repetitive partial/complete collapse of the pharynx during sleep, which results in apnea/hypopnea leading to arterial oxygen desaturations and arousals. Repetitive apnea/hypopnea-arousal episodes cause hypoxia/reoxygenation cycles, which increase free radical generation and oxidative stress that cause motor/sensory nerve impairments and muscle damage. We hypothesize that antioxidants may protect and/or reverse from oxidative stress-induced damage in OSA patients. To understand the acute protective effects of antioxidants on respiratory muscles, we studied the systemic effects of a membrane permeable superoxide dismutase mimetic, Tempol, on genioglossus (EMG_GG_) and diaphragmatic (EMG_DIA_) electro-myographic activities, hypoglossal motoneuron (HMN) nerve activity and cardiorespiratory parameters (mean arterial blood pressure, heart rate) in adult isoflurane-anesthetized obese Zucker rats (OZR) and age-matched lean Zucker rats (LZR). Tempol dose-dependently (1–100 mg/kg) increased EMG_GG_ without changing EMG_DIA_ in OZR and LZR. Tempol increased respiratory rate and tidal volume in OZR and LZR. Tempol (1–25 mg/kg) dose-dependently increased HMN nerve activity in healthy Sprague Dawley rats. Tempol (100 mg/kg) increased EMG_GG_ output by 189% in OZR and 163% in LZR. With respect to mechanisms of effect, Tempol (100 mg/kg) did not augment EMG_GG_ after bilateral HMN transection in Sprague Dawley rats. Although future studies are warranted, available data suggest that in addition to its antioxidant and antihypertensive properties, Tempol can selectively augment EMG_GG_ through modulating HMN and this effect may prevent collapsibility and/or improve stability of the upper airway pharyngeal dilator muscles during episodes of partial and/or complete collapse of the upper airway in OSA human subjects.

## Introduction

Obstructive sleep apnea (OSA) is a common sleep breathing disorder characterized by repetitive episodes of partial (hypopnea) or complete (apnea) obstructions of the upper airway during sleep, despite ongoing efforts to breathe, which leads to decreased blood oxygenation and fragmentation of sleep (Banno and Kryger, 2007; Dempsey et al., 2014; Javaheri et al., 2017). The severity of OSA is determined by apnea-hypopnea index (the total number of apneas and hypopneas events/hour during sleep). In severe cases, respiratory events can occur more than 100 times per hour and typically each event lasts for 20–40 s (Jordan et al., 2014). Clinically, OSA disorder is determined to be present when greater than five abnormal breathing disturbances (apnea-hypopnea events per hour) occur in combination with daytime symptoms of excessive sleepiness, which affects 2–3% of children, 3–7% of middle-aged adults and 10–15% of the elderly population (Jordan et al., 2014). In addition to the obvious detrimental effects of sleep disordered breathing and neurocognitive impairments, OSA is a well-known public-health issue due the high prevalence of severe co-existent cardiovascular morbidities such as systemic and pulmonary hypertension, congestive heart failure, cardiac arrhythmias, atherosclerosis, ischemic heart failure, stroke and silent cerebral infraction (Banno and Kryger, 2007; Dempsey et al., 2014; Javaheri et al., 2017; Yeghiazarians et al., 2021).

The mammalian tongue is mechanically and functionally complex structure with unique intrinsic and extrinsic muscles fibers that collectively produce well-orchestrated muscle movements for rapid alternate between different system requirements for respiration, speech, swallowing and upper airway protection (Ludlow, 2011; Ludlow, 2015). The tongue muscles in mammals are extensively innervated by axons of the hypoglossal motoneurons through medial and lateral branches of the hypoglossal nerves. The hypoglossal motoneurons in turn receive direct and/or indirect excitatory and inhibitory synaptic inputs from brain structures that depend on the activity of the central nervous system, with drive during wakefulness being reduced during sleep and increased during exercise. During non-rapid eye movement sleep, excitatory neuromodulatory inputs provided by noradrenergic, serotonergic and cholinergic neurons decrease while during REM sleep there is direct synaptic inhibition of hypoglossal motoneuron activity by glycinergic inhibitory postsynaptic currents (see Fregosi and Ludlow, 2014). The discharge pattern of hypoglossal motoneurons and the activity of genioglossus muscles are strongly respiratory-dependent (see Fregosi and Ludlow, 2014). Hypoglossal motoneurons tend to be strongly but not exclusively inspiratory modulated. Human subjects do show phasic inspiratory discharge, but many of these units show mainly tonic discharge, with an increase in their firing rate during the inspiratory phase. Increase in respiratory drive with hypercapnia and hypoxia increases hypoglossal motoneuron discharge frequency (see Ludlow, 2011; Fregosi and Ludlow, 2014; Ludlow, 2015).

The intermittent partial or complete obstruction of the upper airway during sleep characteristically leads to a spectrum of mild to severe intermittent hypoxic-hypercapnic/normoxic events resulting in alveolar hypoventilation with nadir hemoglobin oxygen saturations potentially reaching the lower limits (50–60%) of oximeters. These repeated episodes of hypoxia-hypercapnia/normoxia that OSA patients experience resemble re-occurring ischemia/reperfusion events that occur in numerous pathological conditions, and which are known to directly lead to increased circulating markers of oxidative stress and inflammation (Lavie and Lavie, 2009; Eisele et al., 2015). Numerous studies have found that humans with OSA (Christou et al., 2003; Banno and Kryger, 2007; Eisele et al., 2015), animals models of OSA (Dumitrascu et al., 2013) and *in vitro* cell culture models (Prabhakar and Kumar, 2004) exposed to repeated episodes of hypoxia/hypercapnia followed by return to normoxia have substantially higher levels of oxidative stress markers and reduced antioxidant capacity, further supporting the probability that oxidative stress mediates damage to numerous physiological systems in OSA subjects. In particular, oxidative stress is now increasingly recognized as an important contributor to OSA-associated neural injury, cognitive impairment, cardiovascular morbidities, and metabolic derangements (Christou et al., 2003; Kheirandish-Gozal et al., 2014; Eisele et al., 2015; Lavie, 2015).

Apart from oxidative stress, obesity is one of the major known risk factors for OSA (Partinen, 1995); Gottlieb and Punjabi, 2020) and is associated with upper airway narrowing in all age and gender groups (Horner et al., 1989; Schwab et al., 2015). The obese Zucker rat is an established model of genetic obesity and the mechanical characteristics of its pharyngeal airway are similar to those reported in humans with OSA (Farkas and Schlenker, 1994). Compared to lean Zucker rat littermates, the pharyngeal airway is narrower and the upper airway collapsibility, as measured by critical airway pressure, is significantly increased in obese Zucker rats (Magalang et al., 2003; Brennick et al., 2014). Using this animal model with clinical pathophysiology and symptoms of OSA, we used the cell permeable superoxide dismutase-mimetic, Tempol (4-hydroxy-2,2,6,6-tetramethylpiperidine-N-oxyl) (Wilcox and Pearlman, 2008; Wilcox, 2010; Kim et al., 2016) to understand the acute effects of oxidative stress on upper airway dilator muscle activity in spontaneously breathing obese Zucker rats in comparison to age-matched lean Zucker rats. The major finding was that systemic administration of Tempol in obese and age-matched lean Zucker rats significantly and dose-dependently increases the upper airway pharyngeal dilator muscle activity without significantly changing diaphragmatic muscle activity.

## Materials and Methods

### Ethics Statement

All of the rat studies were performed in accordance with the National Institutes of Health Guide for the Care and Use of Laboratory Animals (NIH Publication No. 80.23) revised in 1996. The protocols were approved by the Institutional Animal Care and Use Committee (IACUC) at Galleon Pharmaceuticals, Inc.

### Animals

Age-matched adult male lean (*Lepr* ^fa^/*Lepr* ^+^ or *Lepr* ^+^/*Lepr* ^+^) and obese (*fa*/*fa*) Zucker rats (10–12 weeks old) obtained from Harlan Laboratories (Harlan Laboratories, Indianapolis, IN) were used in this study. Experiments were performed on 16 obese male Zucker rats (mean ± SEM of body weights of 978 ± 31 g) and 16 age-matched lean male Zucker rats (mean ± SEM of body weights of 526 ± 52 g) and eight male Sprague Dawley rats (mean ± SEM of body weights of 338 ± 4 g). The rats were caged individually in an Innocage IVC rat caging system (InnoVive, San Diego, CA, United States) with standard housing conditions with free access to food and water. The vivarium temperature (22°C), humidity (35–40%) and light-dark cycle (12:12 h) were maintained consistently.

### Anesthesia

All surgical procedures were performed under isoflurane anesthesia (2–2.5%) in medical grade air. Following the onset of surgical levels of anesthesia, as judged by abolition of the pedal withdrawal and corneal blink reflexes, the abdomen and neck regions were shaved and cleaned with 70% alcohol and the antiseptic-germicide solution, Betadine surgical scrub (7.5% povidone-iodine, Purdue products, Stamford, CT, United States). Sterile 1% chloramphenicol ointment (Vetcom, Upton, PQ, Canada) was applied to the cornea to prevent drying. A rodent anesthesia mask was placed over the snout, and the rats were spontaneously breathing 2% isoflurane in medical grade air for the remainder of the surgery.

### Effects of Tempol on Cardiorespiratory Parameters and Respiratory Muscle Activities

The effects of Tempol on genioglossus EMG (EMG_GG_), diaphragmatic EMG (EMG_DIA_) and cardio-respiratory parameters were studied in obese (*n* = 6) and age-matched lean Zucker rats (*n* = 6) as described previously (Golder et al., 2008; Baby et al., 2021a). In both obese (*n* = 2) and lean (*n* = 2) Zucker rats saline (ml/kg) was administered repeatedly as a time control. Briefly, with the rats in the supine position, the femoral vein and artery were cannulated using PE-50 tubing (Intramedic, Becton & Dickinson, Franklin Drive, NJ, United States). The femoral arterial catheter was connected to a fluid filled piezoresistive physiological pressure transducer (SP844, AD Instruments, Inc. Bella Vista, Australia). The venous catheter was connected to a 3-way connector for fluid support (4 ml/kg/h, IV; 50:50 mixture of lactated Ringer’s and 6% hetastarch throughout the experiment) and administration of vehicle or Tempol. Two insulated, multi-stranded stainless-steel electrodes (MLA1203, AD Instruments, Inc.) were implanted bilaterally into the tongue musculature via a per-oral approach to record tongue muscles for recording EMG_GG_. To record diaphragm EMG, two insulated, multi-strand stainless steel wires (MLA1203, AD Instruments, Inc.) were implanted onto the side of the diaphragm as it close to the abdominal-wall) via an abdominal approach. The size, configuration, and placement of the electrodes on genioglossus muscle and diaphragm were consistent across experiments. To measure respiratory parameters (respiratory rate, tidal volume, minute ventilation), the cervical trachea was cut, and an endotracheal tube (13G, 2.4 mm OD 1.6 mm ID, Instech Solomon, Plymouth Meeting, PA, United States) was placed into the caudal tracheal stub. The tracheal tube was connected to the pneuomotachometer (differential pressure transducer with 1 L flow head for the measurement of respiratory flow rates; MLT1L, AD Instruments, Inc.). After completion of the surgeries, the rats were allowed to breathe 1.5% isoflurane in medical grade air for the rest of the experiment. After obtaining stable baselines for EMG_GG,_ EMG_DIA_ and cardiorespiratory parameters, Tempol (1, 10, 25, 50 and 100 mg/kg, IV) was administered systemically. In the cumulative bolus paradigm, between each dose, at least 10–20 min were allowed cardiorespiratory parameters to recover to the baseline. Respiratory muscle activities (EMG_GG_ and EMG_DIA_) were also allowed to be stable prior to the administration of each dose of Tempol in lean and obese Zucker rats.

### Effects of Bilateral Hypoglossal Nerve Transection on Tempol-Induced Upper Airway Muscle Activities

Tempol was administered following bilateral hypoglossal nerve (HMNx) transection or Sham surgery to understand the role of HMN in Tempol-induced effects on upper airway muscle function. At the cervical level, HMN was exposed ventrally and bilaterally transected in transected group (*n* = 4). In sham animals, the HMN nerves were exposed but not cut (*n* = 4). After obtaining a stable baseline recording, EMG_GG_ and cardiorespiratory parameters were recorded before and following administration of Tempol (100 mg/kg, IV) in lean Zucker rats.

### Effect of Tempol on Hypoglossal Nerve Activity

To determine the physiological mechanism of action of the Tempol-induced increases in EMG_GG_, hypoglossal motoneuron (HMN) nerve activity was recorded before and after injection of Tempol. HMN output was recorded in isofurane-anesthetized, vagotomized, neuromusculalry paralyzed and mechanically ventilated Sprague-Dawley rats before and after injection of Tempol (1, 10 and 25 mg/kg, IV, *n* = 4) or saline (*n* = 4, time control) as described previously (Golder et al., 2008). Repeated saline injections were used as time control to monitor HMN nerve activity in a control group of rats. Briefly, anesthesia was induced by isoflurane as described above, the cervical trachea was cannulated, and rats were mechanically ventilated (Rodent Ventilator, CWE Inc., Admore, PA). The HMN was isolated, and motor activity was recorded continuously using a bipolar platinum electrode. The HMN action potentials were amplified (NeruroAmp, AD Instruments, Australia) and these amplified signals were rectified, moving time averaged, digitized, recorded and analyzed (PowerLab, LabChart-7Pro software, AD Instruments). Both cervical vagi were transected to prevent vagal sensory feedback that would entertain hypoglossal nerve activity with the ventilator. End-tidal CO_2_ (ETCO_2_) and pO_2_ (ETO_2_) were measured continuously (Capnometer, CWE Inc., PA). After the surgeries were completed, the rats were transferred to urethane (Sigma-Aldrich, St. Louis, MO) anesthesia (1.8 g/kg, IV) while isoflurane was discontinued, and neuromuscularly paralyzed with pancuronium bromide (1 mg/kg, IV; Tocris, Ellisville, MI). At least 30–45 min elapsed after transferring the animals to urethane before IV administration of Tempol or saline. The mechanical ventilator settings (rate or tidal volume) were gradually adjusted to remove the inspiratory activity on the HMN recordings. The ETCO_2_ value at which the inspiratory activity completely absent was referred to as apneic threshold. The baseline nerve activity was established at 1–2 mmHg above the apneic threshold. Peak integrated HMN burst amplitude was recorded for 30 min at baseline conditions and for another 60 min after Tempol or saline administration.

### Data Analyses

The data were averaged in consecutive 15 s time bins for ventilatory (respiratory rate, tidal volume, minute ventilation) and cardiovascular (HR, DBP, SBP and MAP) parameters. The EMG_GG_ and EMG_DIA_ signals were averaged breath by breath and signals were analyzed from the respective moving-time average signals (above electrical zero) and were quantified in percentage change from the preceding baseline for each dose of saline or Tempol. Tonic activity of the HMN, EMG_GG_ and EMG_DIA_ was quantified as mean basal activity. Average values of total amplitude, inspiratory and tonic activities of HMN, EMG_GG_ and EMG_DIA_ were calculated and compared with saline and treatment for efficacy of Tempol on HMN nerve activity, EMG_GG_ and EMG_DIA_. Each rat served as its own control with all interventions performed in one experiment, which allowed for consistent experimental condition (e.g., injection of saline or Tempol) within and between rats. Respiratory, cardiovascular, EMG_GG_ and EMG_DIA_ parameters were expressed as percent of baseline. The percent change in minute ventilation and MAP was plotted against the log dose of Tempol to calculate an ED_50_ value using a variable slope (four-parameters) nonlinear regression model [Y = Bottom + (Top − Bottom)/(1 + 10^(LogED50−X)*HillSlope^)] (Prism; GraphPad Software, Inc., United States). Effects of Tempol on HMN nerve activity, EMG_GG_, EMG_DIA_, respiration and cardiovascular parameters were evaluated by two-way (repeated) measures ANOVA followed by Tukey, Sidak or Bonferroni multiple comparisons tests to determine potential differences between means (Wallenstein et al., 1980). Effects of Tempol on EMG_GG_, EMG_DIA_, ventilatory and cardiovascular parameters after bilateral HMN transection were evaluated by unpaired nonparametric *t-tests*. Differences between means were considered significant when the *p* value was <.05. Statistical analyses were performed using Prism (GraphPad Software, Inc.). All values are expressed as mean ± SEM.

## Results

### Tempol Dose-Dependently and Selectively Increased Genioglossus Muscle Activity

**FIGURE 1:**
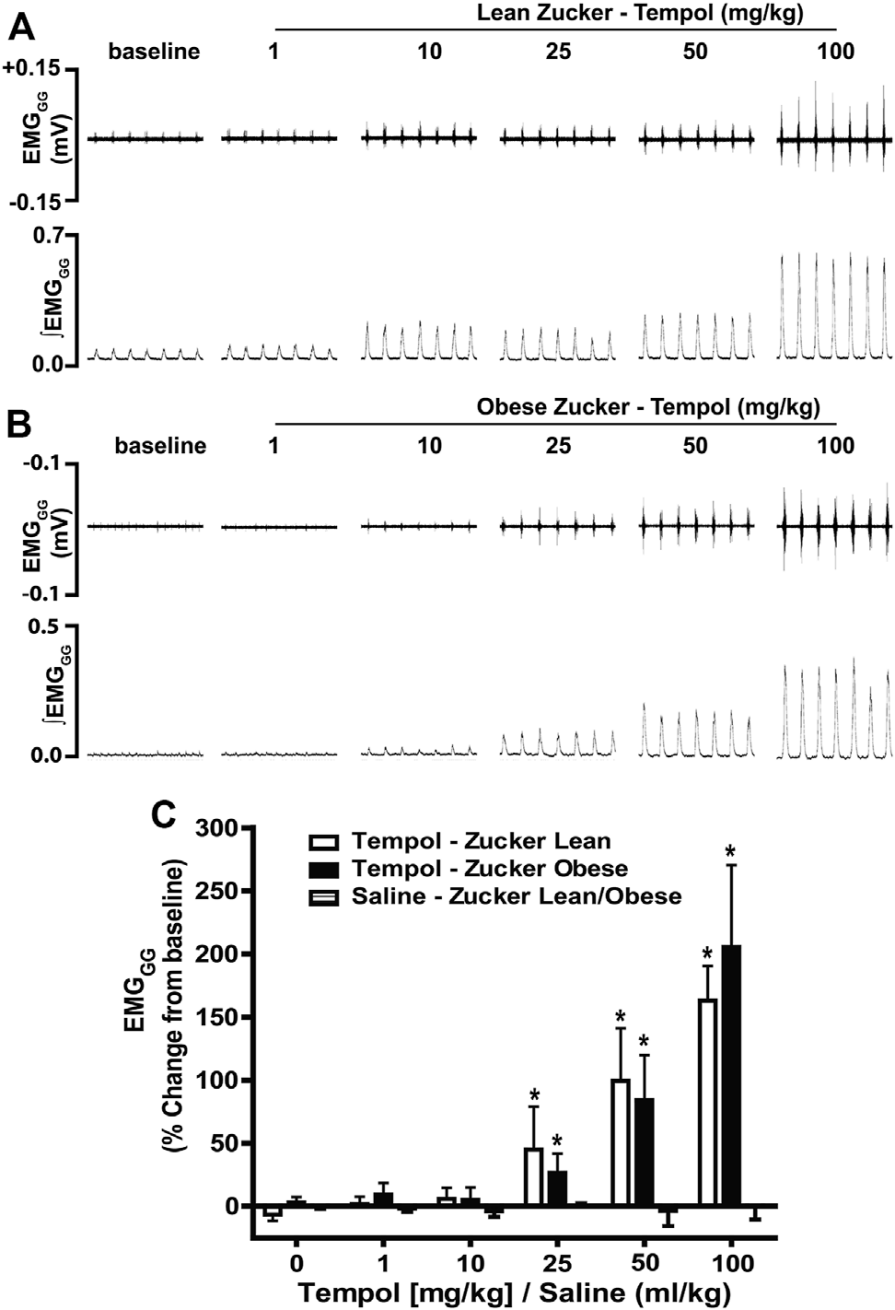
Tempol dose-dependently increased genioglossus muscle activity (EMG_GG_) in obese and lean Zucker rats. **(Panels A and B)**: Representative recordings of raw and integrated EMG_GG_ activities from isoflurane anesthetized spontaneously breathing obese **(A)** and age-matched lean **(B)** Zucker rats respectively, showing the cumulative dose-dependent effects of Tempol (1–100 mg/kg). **(Panel C)**: Percentage changes in EMG_GG_ total amplitude during administration of Tempol in obese (filled bars) and lean (open bars) Zucker rats. Repeated administration of saline (time control) had no effects on EMG_GG_ total amplitude in obese Zucker rats (shaded bars). Values are presented as mean ± SEM. There were six obese Zucker rats and six lean Zucker rats that received Tempol and four lean/obese rats that received saline as a time control. *p < .05, different from saline controls (two-way ANOVA with Sidak multiple comparison tests). There were no differences in the responses elicited by Tempol in the obese and lean Zucker rats (*p* > .05, for all comparisons; two-way ANOVA with Sidak multiple comparison tests).

**FIGURE 2:**
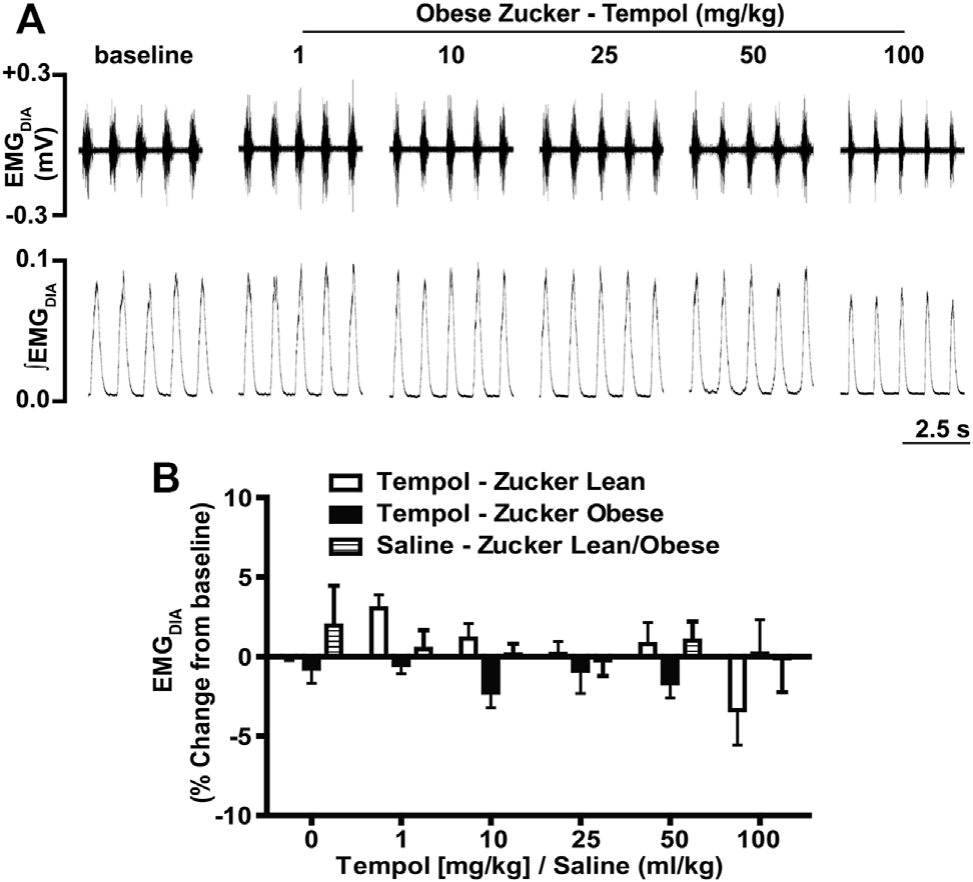
Tempol had no effects on diaphragm muscle activity (EMG_DIA_) in obese and lean Zucker rats. **(Panel A)**: Representative recordings of raw and integrated EMG_DIA_ activity in an isoflurane-anesthetized spontaneously breathing obese Zucker rat treated with Tempol (1–100 mg/kg). **(Panel B)**: Percentage change in EMG_DIA_ total amplitude during administration of Tempol (1–100 mg/kg) in obese (filled bars), lean (open bars) and saline-treated (shaded bars) Zucker rats. Repeated administration of saline (time control) had no effects on total amplitude of EMG_DIAG_ in obese and lean Zucker rats. The values are presented as mean ± SEM. There were six obese Zucker rats and six lean Zucker rats that received Tempol and four lean/obese that received saline as a time control. Tempol did not elicit significant responses in EMG_DIA_ in obese and lean Zucker rats (two-way ANOVA with Sidak multiple comparison tests).

In order to establish the effects of Tempol on respiratory muscles in obese and age-matched lean Zucker rats, the total amplitude of genioglossus (EMG_GG_) and diaphragm (EMG_DIA_) muscle activities were recorded before and after injection of Tempol in isoflurane-anesthetized spontaneously breathing animals. When given as cumulative boluses, Tempol produced a large, dose-dependent (1–10–25–50 and 100 mg/kg, IV) increase in EMG_GG_ amplitude in obese Zucker rats (maximum at 100 mg/kg of approximately 189%) and in age-matched lean Zucker rats (maximum at 100 mg/kg of approximately 163%) (Figure 1). At least 10–15 min were allowed between each dose of Tempol to recover cardiorespiratory parameters and also to have stable recording of respiratory muscle activities The finding that the Tempol-induced increases in EMG_GG_ were comparable in obese and lean Zucker rats (Figure 1) suggests that the mechanisms involved in augmentation of EMG_GG_ was independent of its antioxidant properties (Wilcox, 2010). In contrast to its effects on EMG_GG_, Tempol (1–100 mg/kg, IV) had no effects on the amplitude of EMG_DIA_ in obese or lean Zucker rats (Figure 2). Repeated administration of saline had no effects on EMG_GG_ (Figure 1) or EMG_DIA_ (Figure 2) burst amplitude in lean or obese Zucker rats.

### Cardiorespiratory Effects of Tempol in Obese and Lean Zucker Rats

**FIGURE 3:**
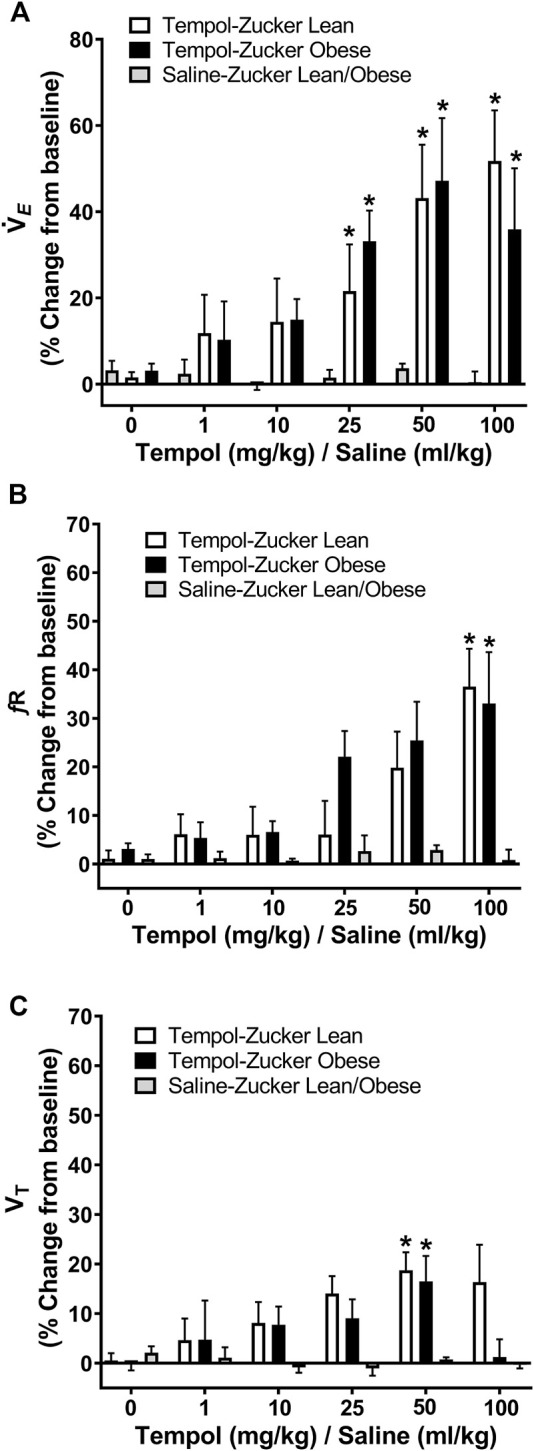
Tempol enhanced breathing in isoflurane-anesthetized obese and lean Zucker rats. Ventilatory parameters were measured by direct tracheal spirometry. Cumulative bolus doses of Tempol (1–100 mg/kg, IV) were administered in lean (open bars) and obese (filled bars) Zucker rats. Peak changes in minute ventilation, frequency of breathing and tidal volume was expressed as percentage change from baseline. Tempol dose-dependently increased minute ventilation **(Panel A)** mostly by its effects on respiratory frequency **(Panel B)** and partially by its effects on tidal volume **(Panel C)**. Repeated administration of equivalent volume of saline (time control, shaded bars) had no effects on of these parameters in lean and obese Zucker rats **(Panels A–C)**. The values are presented as mean ± SEM. There were six obese Zucker rats and six lean Zucker rats that received Tempol and both obese (*n* = 2) and lean (*n* = 2) Zucker rats that received *saline as a time control*. *p < .05, different from saline controls (two-way ANOVA with Sidak multiple comparison tests). There were no differences in the responses elicited by Tempol in the obese and lean Zucker rats (*p* > .05, for all comparisons; two-way ANOVA with Sidak multiple comparison tests).

The ventilatory responses elicited by Tempol were studied by direct tracheal spirometry in anesthetized spontaneously breathing Zucker obese and lean rats. Tempol was not a strong ventilatory modulator in these rats (Figure 3). More specifically, the cumulative administration of Tempol (1–100 mg/kg) elicited moderate, transient and dose-dependent increases in minute ventilation in obese (average maximum approximately 47%) and lean (average maximum approximately 60%) Zucker rats, which were driven mostly by increases in respiratory frequency but also partially by increases in tidal volume (Figure 3). Tempol-induced effects on systemic blood pressure and heart rate we recorded in obese and lean Zucker rats were consistent with and comparable to its known hypotensive actions in other rat strains (Wilcox and Pearlman, 2008).

**FIGURE 4:**
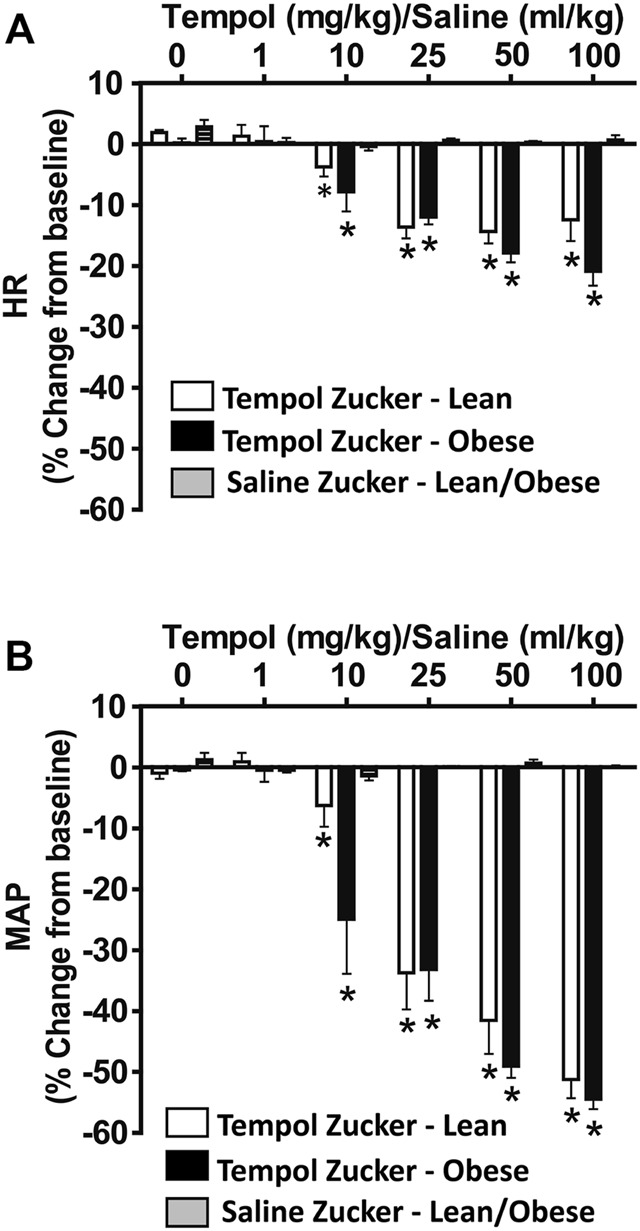
Tempol elicited dose-dependent decreases in heart rate (HR) and mean arterial blood pressure (MAP) in isoflurane-anesthetized animals that were similar in magnitude in obese and lean Zucker rats. HR and MAP were calculated from the arterial blood pressure waveforms in lean (open bars) and obese (filled bars) Zucker rats. Cumulative bolus doses of Tempol (1–100 mg/kg, IV) were given to lean and obese Zucker rats. Tempol dose-dependently decreased HR **(Panel A)** and MAP **(Panel B)** in lean and obese Zucker rats. Repeated administration of saline (time control, shaded bars) had no effects on HR and MAP in lean and obese Zucker rats. Values are presented as mean ± SEM. There were six rats in each of the obese and lean Zucker groups that received Tempol whereas both lean (*n* = 2) and Obese (*n* = 2) Zucker rats received saline. *p < .05, different from saline controls (two-way ANOVA with Sidak multiple comparison tests). There were no differences in the responses elicited by Tempol in the obese and lean Zucker rats (*p* > .05, for all comparisons; two-way ANOVA with Sidak multiple comparison tests).

**FIGURE 5:**
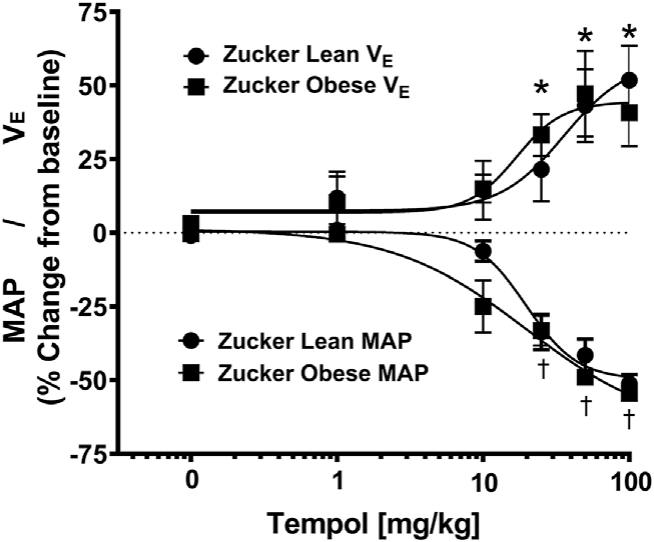
Tempol elicited dose-dependent increases in minute ventilation and decreases in mean arterial blood pressure (MAP) that were similar in magnitude in obese and lean Zucker rats. The maximum changes in minute ventilation (*Y*-axis, positive Y values, 0–75) and MAP (*Y*-axis, negative Y values, 0–75) elicited by cumulative doses of Tempol (1–100 mg/kg, IV) in lean (circle) and obese (square) Zucker rats. Nonlinear regression analysis was performed to identify the ED_50_ values for minute ventilation and MAP in lean and obese Zucker rats. Comparable ED_50_ values for MAP was obtained for lean (19.8 mg/kg) and obese (20.0 mg/kg) Zucker rats. In contrast, ED_50_ values for minute ventilation in obese Zucker rats (15.3 mg/kg) were lower than in the lean Zucker rats (35.9 mg/kg). The values are presented as mean ± SEM. There were six rats in each group. *p < .05, minute ventilation significant change from baseline. ^†^ *p* < .05, MAP significant change from baseline.

As summarized in Figure 4, systemic administration of Tempol (1–100 mg/kg, IV), elicited dose-dependent and transient decreases in heart rate and mean arterial blood pressure (MAP) of comparable magnitude in obese and lean Zucker rats. Repeated administration of saline (time control) had no effects on cardiorespiratory parameters (see Figures 3, 4). Moreover, the Tempol-induced effects on minute ventilation and MAP were comparable in obese and lean Zucker rats (Figure 5). The effective dose at 50% maximum (ED_50_) values for the increase in minute ventilation in obese rats (15.3 mg/kg) was substantially lower than in lean rats (35.9 mg/kg) whereas the ED_50_ values for the decreases in MAP were comparable in lean (19.8 mg/kg) and obese (20.0 mg/kg) Zucker rats.

### Bilateral Hypoglossal Nerve Transection Eliminates Tempol-Induced Effects on Genioglossus Muscle Activity

**FIGURE 6:**
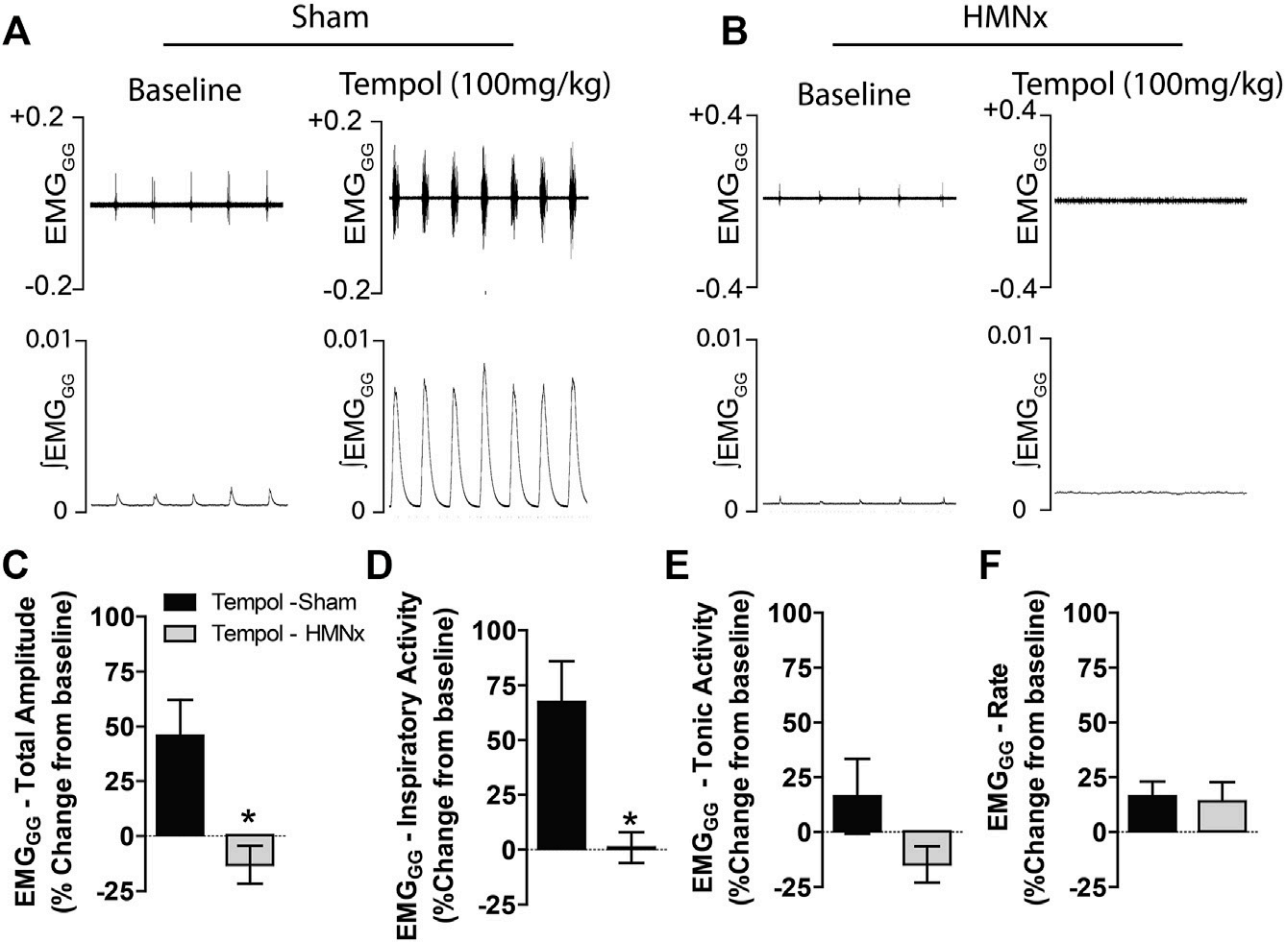
Bilateral hypoglossal nerve transection (HMNx) abolished Tempol-induced effects on genioglossus muscle activity (EMG_GG_). Representative recording showing that the bolus injection of Tempol (100 mg/kg, IV) elicited pronounced increases in EMG_GG_ total amplitude and inspiratory activity in sham-operated rats **(Panel A)** but not in rats after bilateral HMNx **(Panel B)**. Summary data showing that Tempol (100 mg/kg, IV) increased total amplitude **(Panel C)** and inspiratory activity **(Panel D)** in sham-operated but not in HMNx rats. Tempol (100 mg/kg, IV) did not affect tonic activities **(Panel E)** or burst frequency (rate) **(Panel F)** in both groups. Values are presented as mean ± SEM. There were six rats in each group. *p < .05, significant change from baseline.

Having demonstrated that Tempol dose-dependently and selectively increased EMG_GG_ amplitude in obese and lean Zucker rats, we investigated the contribution of the hypoglossal motoneuron pool in the effects of Tempol on EMG_GG_ activity before and after the acute bilateral transection of the hypoglossal nerves (HMNx) or sham-surgery in isofurane-anesthetized spontaneously breathing lean Zucker rats. As summarized in Figure 6, the increases EMG_GG_ activity elicited by Tempol (100 mg/kg) in the sham-operated rats were absent in those with bilateral HMNx. More specifically, HMNx eliminated the Tempol-induced increases in total amplitude, inspiratory and tonic activities of the EMG_GG_.

### Tempol Increases Hypoglossal Motoneuron Output

**FIGURE 7:**
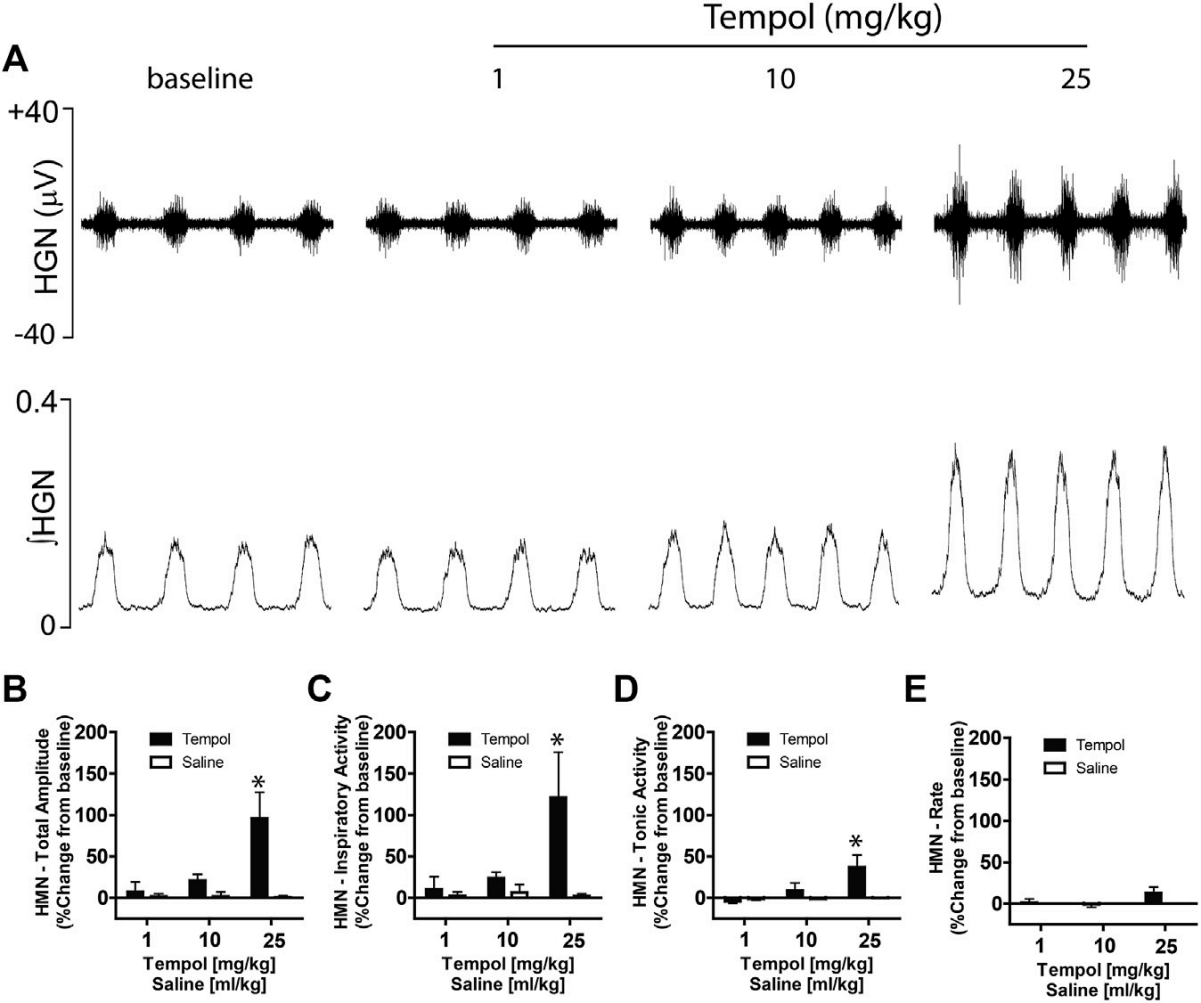
Tempol dose-dependently increased hypoglossal motoneuron (HMN) output. Representative recording showing that Tempol elicited dose-dependent (1, 10 and 25 mg/kg, IV) increases in HMN output in anesthetized, neuromuscularly paralyzed and mechanically-ventilated Sprague-Dawley rats **(Panel A)**. Summary data demonstrating that Tempol-induced dose-dependent increases in total amplitude **(Panel B)**, inspiratory activity **(Panel C)** and tonic activity **(Panel D)** without significant change in HMN burst frequency **(Panel E)**. Repeated administration of saline (time control) had no effects on HMN output **(Panels B–E)**. Values are presented as mean ± SEM. There were four rats in each group. *p < .05, significant change from baseline.

Having demonstrated that bilateral HMN transection abolished the Tempol-induced effects on EMG_GG,_ we speculated that Tempol may augment EMG_GG_ activity by modulating the HMN pool output from the brainstem. To investigate this possibility, we recorded HMN nerve activity before and after administration of Tempol or saline (time control) in anesthetized, mechanically-ventilated and neuromuscularly paralyzed Sprague-Dawley rats. In addition to excitatory and inhibitory neurotransmitters, multiple factors influence HMN output to upper airway pharyngeal dilator muscles (Golder et al., 2008; Grace et al., 2013). Respiratory drive from the central pattern generator, vagal feedback within the brainstem and arterial blood gases (PO_2_ and PCO_2_) are a major determinant of EMG_GG_ activity. Therefore, in order to better evaluate the effects of Tempol while performing the experiments it was essential to maintain stable arterial PaCO_2_ and PaO_2_ levels in the rats by bilateral vagotomy, neuromuscular paralysis using pancuronium bromide (1 mg/kg) and mechanical ventilation. As shown in Figure 7A, Tempol dose-dependently (1–10–25 mg/kg, IV) increased HMN nerve activity whereas injections of saline (vehicle) were without effect. Furthermore, burst to burst analysis of HMN action potentials revealed a dose-dependent increase in total amplitude, inspiratory and tonic activities of HMN output (Figures 7B–D). No changes in HMN rate with Tempol treatment (Figure 7E). Arterial blood gas chemistry (pH, PaCO_2_, PaO_2_) and end tidal gases (ETCO_2_ and (ETO_2_) were tightly regulated during the course of the experiment as these parameters modulate the HMN nerve activity. (Table 1). These data suggest that Tempol augments EMG_GG_ activity predominantly by activating the HMN pool within the brainstem, in addition to its known antioxidative and antihypertensive properties.

**TABLE 1:** Arterial blood-gas chemistry and end tidal values of CO_2_ and O_2_ before and at the end of the completed experimental protocols.

Variables	Baseline	Post-experiment
pH	7.33 ± 0.02	7.31 ± 0.01
PaCO_2_ (mmHg)	52.6 ± 2.1	47.6 ± 3.2
PaO_2_ (mmHg)	137.2 ± 14.4	137.8 ± 7.2
ETCO_2_ (mmHg)	46.8 ± 3.8	46.7 ± 3.6
ETO_2_ (mmHg)	91.6 ± 13.3	92.5 ± 12.7

The data are presented as means ± SEM. There were seven rats in the group. There were no significant changes in these values between baseline and post-experiment (*p* > .05, for a comparisons).

## Discussion

The Tempol-induced effects on respiratory muscles and cardiorespiratory parameters were studied in lean Zucker rats and in age-matched obese Zucker rats, an animal model that has many features related to human OSA phenotypes (Farkas and Schlenker, 1994; Brennick et al., 2014). Systemic administration of Tempol dose-dependently (1–100 mg/kg, IV) and selectively increased EMG_GG_ activity, the major upper airway pharyngeal dilator muscle. The selectivity and efficacy of Tempol in augmenting EMG_GG_ over EMG_DIA_ activity was established in both obese and lean Zucker rats by showing that systemic administration increased EMG_GG_ without changing EMG_DIA_. Furthermore, bilateral transection of the hypoglossal nerves abolished the Tempol (100 mg/kg, IV)-induced effects on EMG_GG_ suggesting that Tempol modulates EMG_GG_, in part, by augmenting HMN output. In fact, systemic administration of Tempol dose-dependently (1–25 mg/kg, IV) and significantly increased tonic and inspiratory activities of HMN output. Collectively, these results suggest that Tempol, a membrane permeable superoxide dismutase-mimetic, can augment EMG_GG_ and improve the stability and/or prevent the collapsibility of the upper airway pharyngeal dilator muscles during episodes of partial and/or complete collapse of the upper airway in human OSA subjects if Tempol were to become an clinically-proven drug. The Tempol-induced increases in EMG_GG_ that presumably would drive retraction of the tongue would directly clear the upper airway. Although the patency of the upper airway is usually considered to be maintained by the activity of cervical muscles in the head and neck, there is compelling evidence that the activity of the diaphragm and chest wall have important roles in maintaining upper airway patency (see van de Graaff, 1988; Bellemare et al., 2005). For example, tonic and phasic forces generated by the thorax can improve upper airway patency (see van de Graaff, 1988) and both cervical muscle and the thorax provide caudal traction to the upper airway (see Bellemare et al., 2005). Moreover, caudal movement of the diaphragm and resulting increased lung volume causes traction that is applied on the mediastinal structures and in turn the upper airway leading to stiffening of the airway walls and greater patency of the upper airway (see van de Graaff, 1988; Bellemare et al., 2005).

To our knowledge, this is the first study to investigate the systemic effects of Tempol on HMN nerve activity and EMG_GG_ activities in an animal model that closely relates to the pathophysiology of human OSA patients. Factors influencing the pathophysiology of OSA in an individual are complex and variable (Kimoff et al., 2011; Vasu et al., 2012; Lavie, 2015; Gottlieb and Punjabi, 2020). However, pharyngeal motor activity in patients with severe OSA during wakefulness is sufficient to maintain a patent airway suggesting suppression of cranial motoneuronal activity by state-dependent changes in synaptic input during sleep is the root cause of pharyngeal hypotonia (Dempsey et al., 2014; Grace et al., 2014; Schwab et al., 2015). HMN nerve activity is dysregulated differentially by withdrawal of excitatory inputs and/or recruitment of inhibitory pathways during non-rapid eye movement (NREM) and rapid eye movement (REM) sleep, respectively (Dempsey et al., 2014; Grace et al., 2014; Schwab et al., 2015; Horner et al., 2017). Among the REM sleep-specific processes, muscarinic receptor-mediated cholinergic inhibition of neural activity in the HMN is a powerful inhibitory system since concomitant local stimulation of HMN with excitatory neurotransmitters or by hypercapnia-mediated increase in central respiratory drive were not sufficient to restore pharyngeal motor activity (Dempsey et al., 2014; Grace et al., 2014; Schwab et al., 2015). Whether Tempol modulates cholinergic activity directly and/or indirectly is an open question however, it is important to identify in a human clinical trial the potential of Tempol to restore pharyngeal hypotonia in NREM and REM sleep states, when various neuronal network dynamics attenuate HMN nerve activity.

Previous *in vivo* and *in vitro* studies have shown that chronic pre-treatment of Tempol prevents the development of upper airway pharyngeal muscle weakness as a result of exposure to chronic intermittent hypoxia (Skelly et al., 2010; Skelly et al., 2012), which clearly suggests that oxidative stress pays a major role in the dysfunction of pharyngeal dilator muscles in this model. Indeed, studies in moderate to severe OSA patients and equivalent animal models have shown the prevalence of oxidative stress along with other confounding factors significantly contribute to OSA (Jelic and Le Jemtel, 2008; Eisele et al., 2015; Lavie, 2015). Oxidative stress is characterized by an imbalance of oxidant and antioxidant mechanisms (enzymatic, non-enzymatic) that control cell homeostasis (Jelic and Le Jemtel, 2008; Eisele et al., 2015; Lavie, 2015). Mechanistically, repetitive episodes of apnea/hypopnea-arousal cause cycles of hypoxia-reoxygenation similar to chronic intermittent hypoxia which increases the generation of reactive oxygen species and oxidative stress which elicit detrimental effects on muscle force by decreasing Ca^2+^ sensitivity of myofibrillar contractile proteins or by decreasing membrane excitability (van der Poel et al., 2008). *In vitro* studies on isolated muscle bundles from upper airway pharyngeal dilator muscle of healthy adult Wistar rats found that antioxidants such as N-acetylcysteine, trion and Tempol increased muscle force and muscle performance in the early phase of fatigue trials (Skelly et al., 2010). Similarly, chronic treatment with Tempol ameliorates chronic intermittent hypoxia-induced decreases in sternohyoid muscle force and performance in a rodent model (Skelly et al., 2012) suggesting potential roles of reactive oxygen species and oxidative stress in the dysfunction of pharyngeal dilator muscles that are known to occur in humans with OSA (Eisele et al., 2015; Lavie, 2015).

In addition to oxidative stress, obesity is also strongly associated with OSA (Gottlieb and Punjabi, 2020). Both of these factors are known to impact the size and function of the upper airway in human OSA subjects and obese Zucker rats. In addition to obesity, obese Zucker rats also present with many of the respiratory deficits commonly observed in obese humans, including reduced lung volumes, reduced chest wall compliance, blunted hypoxic ventilatory responses and anatomically altered upper airway muscles (Ray et al., 2007). The upper airway in obese Zucker rat is more collapsible than the age-matched lean Zucker rats and the critical pressure necessary to close the upper airway in obese Zucker rat is substantially lower than in age-matched lean Zucker rats, a condition similar to OSA patients compared to those without OSA (Ogasa et al., 2004). Despite the prevalence of oxidative stress, abnormal upper airway morphology and respiratory insufficiency in obese Zucker rats, Tempol elicited comparable effects on EMG_GG_ in obese and age-matched lean Zucker rats. This finding suggests that a separate pharmacological action of Tempol (rather than its antioxidant properties) is responsible for augmenting upper airway muscle activity (Wilcox and Pearlman, 2008). Moreover, selective inhibition of K^+^ channels in the HMN motoneuron pool increases tonic and respiratory-related EMG_GG_ activities (Grace et al., 2013; Gurges et al., 2021). In contrast, Tempol is a BK-channel agonist and as such must be modulating/increasing HMN activity through separate as yet unidentified mechanisms (Xu et al., 2006; Chen et al., 2007; Wilcox and Pearlman, 2008; Further studies are warranted to understand the non-antioxidant/non-BK-channel-mediated pathway by which Tempol increases HMN nerve and upper airway muscle activities.

The Tempol-induced enhancement of ventilation in lean and obese Zucker rats is another novel finding of this study. The effects of Tempol manifested as an increase in minute ventilation that arose from elevations of tidal volume and respiratory rate was consistent with findings in anesthetized Sprague-Dawley rats (Baby et al., 2021a). The sites and mechanisms by which Tempol increases ventilation need investigation. Previous pre-clinical (Golder et al., 2015) and clinical (McLeod et al., 2014) studies have shown that the pharmacological *inhibition* of large-conductance Ca^2+^-activated K^+^ (BK)-channels in the carotid body increases ventilation and attenuates opioid-induced respiratory depression. In contrast, Tempol is a BK-channel *agonist* (Xu et al., 2006) suggesting that the Tempol-induced increase in ventilation occurs by processes other than modulation of BK-channel activity. Tempol is lipophilic (Wilcox and Pearlman, 2008) and may have elicited its excitatory effects on breathing by actions in peripheral structures such as the carotid bodies and skeletal muscle within the chest wall and diaphragm as well as in central nuclei/structures in the brainstem known to process ventilatory information including the nucleus tractus solitarius (Heistad et al., 1974). In addition, the baroreceptor reflex modulates chemoreceptor reflex-mediated changes in ventilation (Heistad et al., 1974; Anand and Paintal, 1988; Anand, 1996) and there are numerous central and peripheral processes by which changes in arterial pressure affects the magnitude of the chemoreceptor reflex response and many neural stimuli concurrently converge to influence the chemoreceptor response during hypotension (Anand, 1996). In general, the sensitivity of carotid body chemoreceptors to neural stimuli are augmented during hypotension due to increases in the sympathetic nerve activity and the release of catecholamines within the carotid bodies (Heistad et al., 1974). As such, Tempol-induced hypotension may have directly/indirectly participated in the expression of the Tempol-induced increases in minute ventilation in both lean and obese Zucker rats.

The Tempol-induced bradycardia and hypotension was dose-dependent and comparable in lean and obese Zucker rats and consistent with the known anti-hypertensive property of Tempol in hypertensive rat models (Wilcox and Pearlman, 2008) and also as reported in anesthetized Sprague Dawley rats (Baby et al., 2021a). The blood pressure and heart rate responses elicited by the IV administration of Tempol in the obese and lean Zucker rats were transient and recovered to baseline within minutes depending on the dose, as observed in anesthetized Sprague Dawley rats (Baby et al., 2021a). Both acute and prolonged administration of Tempol reduces arterial blood pressure and heart rate in anesthetized and conscious animal models. There are a number of neural and cellular mechanisms that converge together to elicit the hypotensive effects of Tempol (Wilcox and Pearlman, 2008). For example, systemic or local administration of Tempol markedly decreases sympathetic nerve activity by activation of ATP-sensitive K^+^-channels (Shokoji et al., 2004). Moreover, Tempol increases the bioactivity of vascular nitric oxide by reducing the levels of vascular oxygen radicals which enhances nitric oxide-mediated vasodilation (i.e., decreases in peripheral vascular resistance (Wilcox and Pearlman, 2008). Collectively, these studies suggest that Tempol-induced hypotension is due to converging mechanisms including nitric oxide-mediated decreases in peripheral vascular resistance and inhibition of peripheral sympathetic nerve activity by activation of ATP-sensitive K^+^-channels (Shokoji et al., 2004; Wilcox and Pearlman, 2008).

## Study Limitations

This study provides mechanistic insights into how Tempol affects the systems that regulate upper airway function and ventilatory control. An important but we would argue, a necessary limitation, is the use of isoflurane-anesthetized obese and lean Zucker rats and also isofurane-anesthetized, vagotomized, neuromusculalry paralyzed and mechanically ventilated healthy Sprague-Dawley rats. Where possible, it would be preferable to perform these studies in unanesthetized awake and sleeping rats that are chronically-instrumented to examine the effects of Tempol in rats free from anesthesia and paralyzing agents. Another study limitation is the lack of females in the study groups. It is well established that sex is an important factor in many aspects of the processes that generate free radicals/superoxide anion (Tenkorang et al., 2018) and inflammation (Di Florio et al., 2020). We will include female obese and lean Zucker rats of various ages in on-going studies. Positive findings of these new to be completed studies would strengthen the argument for the use of Tempol in human clinical trials related to the development of thereapeutics for sleep-apnea.

## Conclusion

In conclusion, our findings demonstrate that the systemic administration of Tempol selectively modulates EMG_GG_ in obese and age-matched lean Zucker rats by mechanisms that appear to be independent of its ability to act as a superoxide anion scavenger, antioxidant or modulator of BK-channels. The dose-dependent increases in EMG_GG_ activity elicited by Tempol and the attenuation of the effects of Tempol on EMG_GG_ activities after bilateral transection of the hypoglossal nerves raises the possibility that in addition to its known properties, Tempol may act on GG premotor or motor networks to increase hypoglossal motor nerve activity. This study also confirms that Tempol augments ventilation whereas it depresses heart rate and MAP (Baby et al., 2021a). Collectively, data presented here suggesting that Tempol augments EMG_GG_ by modulating hypogossal motor nerve activity raises the possibility that this drug may prevent the collapsibility and/or improve the stability of the upper airway pharyngeal dilator muscles during episodes of partial/complete collapse of the upper airway in OSA patients.

## Data Availability

The raw data supporting the conclusion of this article will be made available by the authors, without undue reservation.

## Abbreviations

BK-channels, large-conductance Ca^2+^-activated K^+^-channels; EMG_DIA_, diaphragmatic EMG; EMG_GG_, genioglossus EMG; HMN, hypoglossal motoneuron; HMNx, bilateral hypoglossal nerve transection; NREM, non-rapid eye movement sleep; OSA, obstructive sleep apnea; REM, rapid eye movement sleep; Tempol, 4-hydroxy-2,2,6,6-tetramethylpiperidine-N-oxyl
